# Emerging insights into mesenchymal stem cells and exosome-based therapies for liver injury

**DOI:** 10.17305/bb.2025.12144

**Published:** 2025-05-05

**Authors:** Shaolong Yang, Juanyu Liu, Jia Siang Khe, Alvin Jiunn Hieng Lu, Vuanghao Lim

**Affiliations:** 1 Advanced Medical and Dental Institute, Universiti Sains Malaysia, Bertam Kepala Batas, Penang, Malaysia; 2 School of Nursing, Zhengzhou Railway Vocational & Technical College, Pengcheng Avenue, Zhengdong New District, Zhengzhou, Henan Province, China; 3 Department of Orthopaedic Surgery, Faculty of Medicine, University of Malaya, Kuala Lumpur, Malaysia; 4 Department of Biomedical Science, Faculty of Medicine, University of Malaya, Kuala Lumpur, Malaysia

**Keywords:** Liver injury, mesenchymal stem cells, MSCs, exosomes, hepatic fibrosis

## Abstract

Hepatic ischemia-reperfusion injury, fatty liver, liver fibrosis, liver peroxidative injury, and drug-induced liver injury are among the most common liver diseases. Mesenchymal stem cells (MSCs) possess multi-lineage differentiation potential and immunomodulatory functions. In the treatment of liver injury, MSCs can promote repair through homing effects, direct differentiation into hepatocyte-like cells (HLCs), immunomodulation, and anti-fibrotic mechanisms. Clinically, MSCs contribute to liver injury repair either directly or indirectly via the secretion of exosomes. Beyond their reparative role, MSC-derived exosomes can also serve as molecular biomarkers for the diagnosis and prognosis of liver diseases. Establishing higher-quality standards, robust auditing and evaluation systems, and conducting deeper mechanistic studies are essential prerequisites for the future clinical application of MSCs in the treatment of liver diseases.

## Introduction

The liver is a vital organ responsible for various essential functions, including metabolism, detoxification, digestion, and immune defence. It plays a critical role in storing glycogen, synthesizing proteins, secreting bile for digestion, and detoxifying harmful substances from the bloodstream. Additionally, the liver serves as an important immune organ by filtering pathogens and toxins, making it crucial for maintaining overall health and homeostasis in the human body [1]. However, the liver is highly susceptible to damage from various factors such as viral infections, chronic alcohol consumption, abnormal lipid metabolism, exposure to drugs and toxins, and autoimmune conditions. Chronic viral infections, particularly hepatitis B (HBV) and hepatitis C (HCV), are leading causes of liver damage globally. Excessive alcohol intake is another major contributor, leading to alcoholic liver disease (ALD), while metabolic disorders such as obesity and insulin resistance are associated with nonalcoholic fatty liver disease (NAFLD).

When these factors persist, they cause inflammation and injury to the liver, disrupting its metabolic and detoxification processes. Over time, this can lead to a range of liver diseases including fibrosis, cirrhosis, and liver failure. In severe cases, liver damage can progress to life-threatening conditions such as end-stage liver failure or hepatocellular carcinoma (HCC), a primary form of liver cancer. These liver diseases represent significant health challenges, contributing to millions of deaths annually worldwide, underscoring the importance of prevention, early detection, and innovative treatments for liver-related disorders [2].

**Table 1:** Comparative analysis of cell sources for hepatic regenerative therapy

**Types of cells**	**Advantages**	**Limitations**	**Ref**
Hepatocyte	Less invasive Less expensive Cryopreserved cells are available when needed Multiple patients can be treated from one donor tissue	Limited source of cells from liver donors Difficult to expand and cryopreserve Immune rejection Poor cell engraftment	[13]
MSCs	No ethical restrictions Easily scalable for transplantation Extend graft survival Immunomodulatory, anti-fibrotic, and regenerative properties	Evidence primarily from clinical studies; challenges in application consistency	[14–16]
ESCs	Extreme self-replication ability High differentiation potential	Ethical concerns surrounding use Risk of tumorigenicity and immune rejection	[17]
iPSCs	Easy derived from any cell source (e.g., Skin fibroblast or mononuclear white blood cell) Extreme self-renew capability and differentiation potential Can perform gene editing to treat genetic disease	Tumorigenicity concerns due to incomplete differentiation Long-term safety, tolerability and efficacy of iPSCs-derived hepatic cell	[18]

iPSC: Induced pluripotent stem cell; ESC: Embryonic stem cell; MSC: Mesenchymal stem cell.

## Current standard therapies for liver disease

Various types of chronic liver disease may eventually progress to liver fibrosis due to persistent liver damage and other contributing factors [3]. Current treatment strategies for liver disease include pharmacological therapy, lifestyle modifications, and surgical interventions. For ALD, preferred treatment options include alcohol abstinence, nutritional support, and lifestyle changes as adjunctive therapies. In severe cases, glucocorticoid therapy and liver transplantation are considered effective [4]. Patients with NAFLD are typically obese and exhibit insulin resistance and/or metabolic syndrome. Therefore, treatment primarily focuses on reducing risk factors through gradual weight loss achieved via lifestyle modifications, emphasizing nutrition and exercise [5]. Additional therapies, such as insulin sensitizers (e.g., thiazolidinediones) and antioxidants (e.g., vitamin E), have also shown clinical benefit [6]. Autoimmune hepatitis (AIH) is commonly treated with corticosteroids such as prednisone or prednisolone. Alternative immunosuppressive agents, including mycophenolate mofetil (MMF), calcineurin inhibitors (e.g., cyclosporine A and tacrolimus), and biologic agents like infliximab, may be used as adjunctive treatments [7]. It is important to note that treatment regimens should be individualized based on the patient’s specific condition and response to initial therapy. Although a variety of pharmacologic agents are used clinically to treat liver disease [8], these therapies often face limitations, including poor organ specificity, limited tissue distribution, and potential for drug-induced toxicity, which constrain their long-term effectiveness. Moreover, drug-related liver injury remains a significant barrier to the clinical use of many medications for liver disease. For patients with end-stage decompensated cirrhosis or acute liver failure, liver transplantation remains the definitive treatment. While it has improved survival rates, transplantation is challenged by a shortage of donor organs, risk of immune rejection, and increased postoperative complications and infections. Given these limitations, there is an urgent need for novel therapeutic strategies that promote liver regeneration and repair. Recent research has highlighted the potential of mesenchymal stem cells (MSCs) and their derived exosomes as regenerative therapies for liver disease. MSCs exhibit immunomodulatory and regenerative capabilities, aiding liver repair through anti-inflammatory, anti-fibrotic, and hepatoprotective mechanisms. Their extracellular vesicles (EVs), known as exosomes, transport bioactive molecules that facilitate intercellular communication and tissue regeneration, offering a promising alternative to conventional treatments. Wu et al. (2022) [9] demonstrated that MSC-derived exosomes (MSC-Exos) can modulate hepatic immune responses by promoting macrophage polarization, inhibiting inflammatory cytokines, and enhancing the liver’s anti-inflammatory microenvironment. Given the distinct pathophysiology of different hepatic disorders, tailored approaches to EV-based therapies are essential, underscoring the importance of individualized MSC and exosome treatments [10].

Exosome research has rapidly evolved, with foundational studies highlighting their diverse roles in disease progression and tissue regeneration. However, the aging and senescence of MSCs may reduce their therapeutic effectiveness. Senescent MSCs can release EVs containing pro-senescent factors [11], which may induce senescent drift in recipient hepatic cells. This process can accelerate tissue dysfunction and fibrosis, posing challenges for MSC-based regenerative therapies. To address these risks, innovative strategies—such as MSC preconditioning or genetic modification—are needed to mitigate the effects of senescent EVs and enhance therapeutic potential while minimizing adverse outcomes. This underscores the importance of further research into MSC- and exosome-based therapies as novel strategies for treating liver diseases.

## Stem cell-based approaches

As a potential alternative to in situ liver transplantation, liver cell transplantation is simpler, less invasive, and relatively safe. Its clinical use has increased in recent years. This treatment involves transplanting cells—obtained through *in vitro* culture or isolation—into the recipient’s liver, where they can proliferate, gradually repair damaged hepatocytes, reconstruct liver architecture, and restore liver function. Recent advances in culture conditions, media composition, and techniques have expanded the range of viable cell sources. In addition to primary isolated hepatocytes, other cell types, such as MSCs, hepatocyte-like cells (HLCs), and organoid-derived cells have demonstrated greater potential for repairing liver damage [12]. The characteristics and advantages of different cell types in hepatocyte transplantation are summarized in Table 1.

### Hepatocytes (primary or induced)

Progenitor or induced hepatocytes can synthesize substances such as albumin and urea, as well as perform detoxification and metabolic functions. After transplantation into a recipient’s liver, primary hepatocytes can colonize the host tissue, continuously proliferate, and partially restore liver functions [19]. These cells have the potential to generate new, healthy tissue and repopulate the liver environment. Fetal liver cells, including hepatic stem/progenitor cells, have demonstrated efficient liver repopulation and differentiation into functional hepatic cells [20]. Additionally, chemically induced liver progenitors (CLiPs), derived from adult rat hepatocytes, can differentiate into both hepatocytes and biliary epithelial cells [21]. These findings suggest that both primary and induced hepatocytes are capable of replacing liver functions and offer promise for cell transplantation therapies in hepatic disorders. However, their clinical application remains limited by the inability of hepatocytes to expand robustly *in vitro* and by the decline in cell viability following cryopreservation and thawing [22].

### Embryonic stem cells (ESCs)

ESC-derived endodermal cells can generate highly enriched hepatocyte precursors, improve liver function, enhance survival in liver failure, support drug testing and disease modeling, and potentially serve as an alternative to liver transplantation for treating liver injury [23]. These cells have been shown to significantly increase survival rates following transplantation into mice with induced hepatocellular injury; however, concerns remain regarding long-term engraftment and the risk of tumor formation [24]. Human ESC-derived hepatic organoids (hEHOs) also show promise for therapeutic liver repopulation and for modeling pathophysiological conditions such as alcoholic liver injury [25]. Notably, hEHOs can be expanded over multiple passages and differentiate into mature hepatocytes without forming teratomas. Additionally, human ESC-derived MSCs and their EVs demonstrate potential in treating liver fibrosis by suppressing inflammation and promoting collagenase activity [26].

### Induced pluripotent stem cells (iPSCs)

iPSCs hold promise as a reliable source of hepatocytes for various medical applications, owing to their unlimited proliferative capacity, genetic diversity, and ethical advantages [27]. iPSCs can differentiate into HLCs that exhibit similar phenotypes and physiological functions [28]. However, achieving efficient and precise differentiation of iPSCs into HLCs remains a significant challenge.

### MSCs

MSCs are derived from various tissues, including bone marrow, umbilical cord, placenta, adipose tissue, peripheral blood, and muscle. They express specific surface markers, such as CD73, CD90, and CD105. As multipotent stem cells, MSCs can differentiate into osteoblasts, adipocytes, and even hepatocytes, making them widely used in research and clinical applications. Clinical studies using bone marrow-derived MSCs have reached phase I/II randomized controlled trials, demonstrating that MSC transplantation can effectively treat liver diseases, such as fibrosis and cirrhosis (Schacher et al., 2021) [29]. Numerous studies support this, reporting positive outcoms for MSC-based treatments of liver conditions [30]. For example, human adipose-derived MSCs (AD-MSCs) transplanted intrahepatically into rats with thioacetamide-induced chronic liver injury differentiated into albumin-expressing hepatocytes within one week [31]. Similarly, porcine-derived MSCs injected into mice with carbon tetrachloride (CCl_4_)-induced acute liver failure significantly reduced serum levels of alanine aminotransferase (ALT), aspartate aminotransferase (AST), direct bilirubin (DBiL), and total bilirubin (TBiL), while increasing albumin (ALB) levels, improving survival and mitigating liver injury [32]. In a D-galactosamine (D-gal)-induced acute liver failure pig model, intravenous transplantation of human BM-MSCs at a dose of 3 × 10^6^ cells/kg effectively alleviated liver failure and promoted *in vivo* differentiation into mature hepatocytes [33]. Furthermore, a meta-analysis involving 854 patients showed that MSC therapy significantly improved liver function markers, including MELD score, TBiL, and ALB levels, compared to conventional treatment. The therapy also increased overall survival rates, with the greatest benefit observed in patients with acute-on-chronic liver failure (ACLF) [34]. Thanks to their ease of access and low immunogenicity, MSCs are the primary stem cells used in clinical liver disease treatment. However, they are not yet approved for clinical use in end-stage liver disease, likely due to their limited ability to differentiate into hepatocytes *in vivo*. This limitation may hinder their capacity to modulate immune responses and suppress inflammatory mediators via paracrine effects. Despite these challenges, MSCs show promise in promoting liver function recovery and modifying the host response to liver injury. Notably, conditioned medium from tonsil-derived MSCs (T-MSC CM) has been shown to alleviate CCl_4_-induced liver fibrosis and inflammation in mice [35].

## MSCS and their exosomes

### MSC biology and sources

MSCs are multipotent stem cells capable of differentiating into various cell types, including osteoblasts, chondrocytes, hepatocytes, and adipocytes [36, 37]. They play a crucial role in repairing damaged or aging tissue and are essential for the regeneration of native functional tissue [38]. In addition to their regenerative capabilities, MSCs possess immunomodulatory properties, which are widely utilized in tissue grafting to help prevent graft rejection [39]. MSCs can be derived from various tissue sources, each offering distinct advantages. Bone marrow is considered the gold standard due to its high potential for bone regeneration, though its extraction is invasive [40]. Adipose tissue provides a higher yield of MSCs and can be harvested through less invasive procedures like liposuction [41]. Umbilical cord MSCs, obtained from Wharton’s jelly—a medical byproduct of childbirth—offer a non-invasive source with a high proliferative capacity, as these cells are younger and exhibit greater regenerative potential [42, 43]. Other sources of MSCs include the placenta, dental pulp, peripheral blood, and synovial fluid. Each source has unique benefits depending on the intended application, such as orthopedic treatments or general tissue regeneration [44, 45].

MSC-Exos have emerged as a promising alternative to direct MSC therapy due to their roles in immunomodulation and fibrosis resolution. However, recent studies suggest that their efficacy may depend on their tissue of origin. For instance, Shi et al. (2022) [46] demonstrated that exosomes derived from umbilical cord MSCs exhibit superior immunomodulatory effects in nonalcoholic steatohepatitis (NASH) compared to those from adipose tissue MSCs, potentially due to enhanced secretion of anti-inflammatory cytokines. In contrast, bone marrow MSC-Exos have been widely studied for their antifibrotic effects in HBV-related cirrhosis, attributed to their ability to downregulate transforming growth factor-β (TGF-β)/Smad signaling and reduce collagen deposition [47]. Meanwhile, adipose tissue MSC-Exos have been investigated for their potential in non-ALD, acting through anti-inflammatory, antifibrotic, and metabolic pathways [48].

### MSC exosomes

Exosomes are nano-sized vesicles that contain distinct proteins, lipids, and nucleic acids. They play vital roles in cellular waste disposal and intercellular communication, with important implications for immune regultion and disease progression. Exosomes can transport functional microRNAs and other small RNAs, contributing to gene regulation in recipient cells [49]. MSC-Exos, in particular, show promise as an alternative treatment for liver diseases due to their small size, low immunogenicity, and lack of tumorigenic risk [50]. These stem cell-derived exosomes exhibit multifaceted therapeutic effects on liver injury: they can promote hepatocyte proliferation, reduce apoptosis, suppress inflammation, and alleviate fibrosis. Moreover, they can be bio-engineered to deliver specific molecules, enhancing their ability to target liver damage more precisely [51].

**Figure 1.**
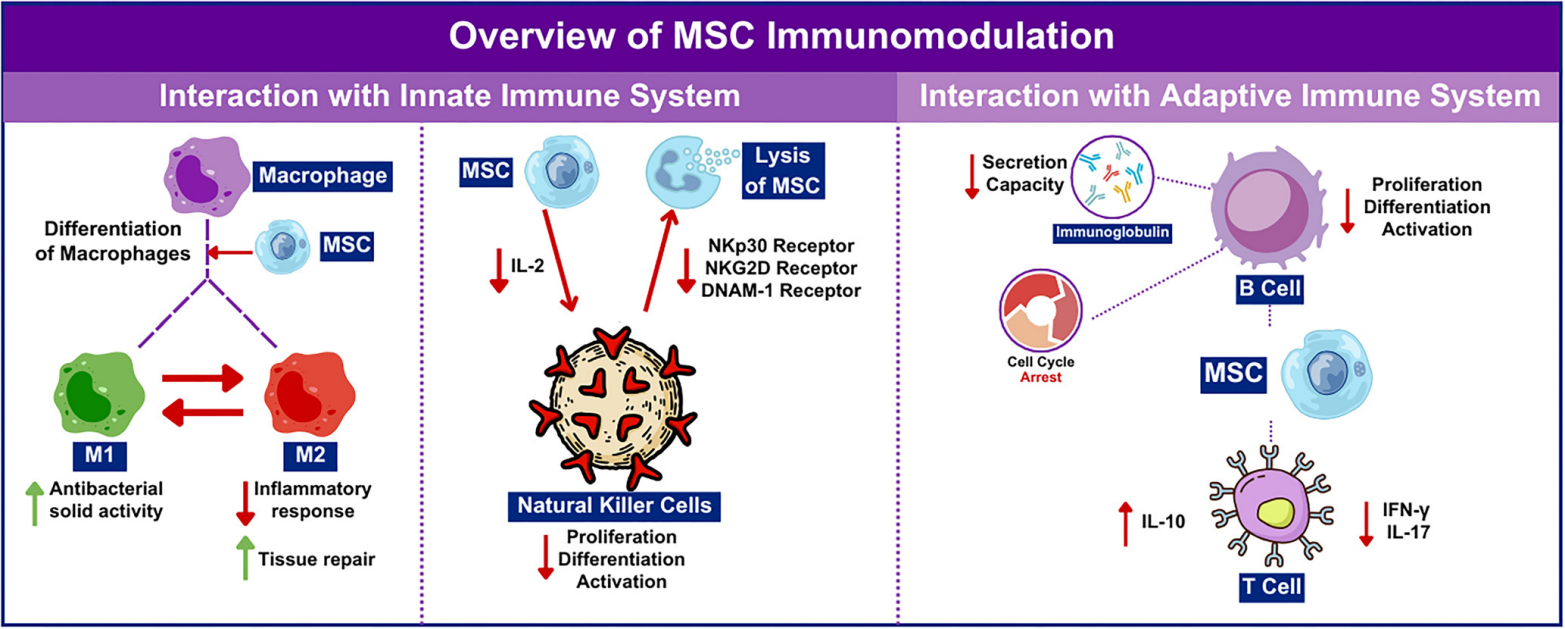
**The overview of MSC immunomodulation**. MSC: Mesenchymal stem cell; IL-10: Interleukin-10.

## Mechanisms of action

### Hepatic differentiation

MSCs are multifunctional stem cells that originate from the mesoderm and ectoderm during early development. They are therapeutically valuable due to their self-renewal capacity and ability to differentiate into various adult cell types, including adipocytes, osteoblasts, chondrocytes, and myogenic cells *in vitro*. Studies have shown that MSCs can also be induced to differentiate into HLCs *in vitro* and express hepatocyte-specific markers [52]. In an experiment by Luk et al. [53], BM-MSCs co-cultured with either normal or damaged hepatocytes—without added growth factors—differentiated into HLCs, suggesting that hepatocyte-secreted factors may drive this transformation. Zheng et al. compared the liver differentiation potential of human amniotic fluid-derived MSCs (AF-hMSCs) and BM-MSCs, finding that AF-hMSCs exhibited greater potential [54]. Interestingly, MSCs that underwent partial differentiation still expressed stem cell markers such as CD90, indicating incomplete transformation. Additionally, Sato et al. [55] observed Y-chromosome-labeled human MSCs in the livers of allyl alcohol-treated rats with acute liver injury, supporting the possibility of hepatocyte differentiation after xenotransplantation.

**Figure 2.**
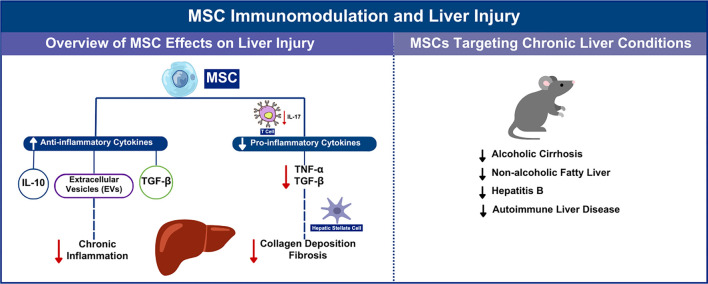
**MSC immunomodulation and liver injury**. MSC: Mesenchymal stem cell; TGF-β: Transforming growth factor-β; IL-10: Interleukin-10.

### Immunomodulatory effect

#### MSC immunomodulatory mechanisms

MSCs possess potent immunomodulatory functions and help maintain homeostasis *in vivo* by secreting soluble factors that interact with both innate and adaptive immune cells. These properties have promising applications in the treatment of liver diseases. Figure 1 provides an overview of the immunomodulatory mechanisms of MSCs. Within the innate immune system, macrophages and neutrophils play crucial roles in inflammation and tissue repair. In inflamed or damaged tissues, MSCs have been shown to promote the differentiation of macrophages into both M1 and M2 phenotypes. M1 macrophages exhibit strong antibacterial activity, while M2 macrophages help resolve inflammation and support tissue repair by secreting trophic factors and interleukin-10 (IL-10), as well as facilitating the clearance of apoptotic cells [56]. NK cells are another key component of the innate immune system. The MSC secretome—which includes various bioactive molecules, such as nucleic acids, cytokines, chemokines, and small molecules—has been found to suppress NK cell activity. Proteomic analyses have identified several secretome components that modulate inflammation and inhibit NK cell effector functions, partly by increasing expression of the inhibitory receptor CD96, which activates inhibitory signaling pathways [57]. Conversely, activated NK cells are capable of lysing MSCs. MSCs can inhibit IL-2-induced NK cell proliferation, yet the interaction can lead to MSC lysis mediated by activating NK receptors, such as NKp30, NKG2D, and DNAM-1, which recognize ligands expressed on MSCs [58]. In the adaptive immune system, MSCs primarily exert inhibitory effects on the proliferation, differentiation, and function of immune cells. MSCs have been shown to modulate T cell differentiation by suppressing the secretion of IFN-γ and IL-17, thereby inhibiting the differentiation of Th1 and Th17 cells. At the same time, MSCs promote IL-10 secretion, which induces regulatory T cell (Treg) production and helps restore the balance between effector and Tregs. B cells, another major component of adaptive immunity, are also modulated by MSCs. Both human and murine MSCs have been shown to inhibit B cell proliferation, differentiation, and activation. B cells co-cultured with MSCs exhibit cell cycle arrest, impaired immunoglobulin production, and reduced chemotactic response [59, 60].

#### MSC immunomodulation and liver injury

MSCs can inhibit chronic inflammation and alleviate liver fibrosis by regulating the proliferation and apoptosis of HSCs, as well as the secretion of TGF-β and collagen deposition [61]. IL-17 can stimulate HSCs to express TNF-α and TGF-β, thereby promoting their activation. Additionally, IL-17 directly induces collagen expression in HSCs and promotes their transformation into fibroblasts through signal transduction and activation of signal transducer and activator of transcription 3 (STAT3) [62]. These findings indicate that IL-17 plays a crucial role in promoting fibrosis and is associated with the severity of liver injury. Recent studies have provided quantitative evidence supporting the efficacy of MSC-Exo therapy in treating liver fibrosis. Zhou et al. (2024) demonstrated that MSC-Exos significantly reduce liver fibrosis in preclinical animal models. Their analysis showed improvements in histopathological scores and biochemical markers of liver function, indicating the effectiveness of exosomes in attenuating fibrotic processes. Furthermore, combining MSC-Exos with other therapeutic agents enhanced their antifibrotic effects, suggesting a synergistic potential for future combination therapies [30]. Additional studies have investigated the use of pretreated MSCs in a mouse model of chronic alcoholic cirrhosis. Pretreating MSCs with lysophosphatidic acid (LPA) or sphingosine-1-phosphate (S1P) significantly enhanced their therapeutic effects, including improvements in tissue damage, oxidative stress, inflammation, fibrosis, and lipid metabolism dysfunction. These effects were accompanied by increased alcohol-metabolizing enzyme activity, which may be linked to elevated IL-10 secretion by pretreated MSCs [63]. In models of NAFLD, MSC treatment reduced proinflammatory cytokine expression, decreased tissue inflammation, and improved liver morphology, confirming their protective effect on liver function. In addition to alcoholic cirrhosis, liver fibrosis can also result from HBV and autoimmune liver diseases. Studies have shown that EVs secreted by BM-MSCs significantly improved liver function in a mouse model of AIH [64]. A randomized controlled clinical trial investigating acute and chronic liver failure due to HBV found that peripheral infusion of allogeneic BM-MSCs was safe and significantly improved patient survival at 24 weeks post-infusion. The improvement was attributed to enhanced liver function and a reduced incidence of serious infections [65]. Figure 2 illustrates the overall immunomodulatory functions of MSCs in liver injury.

**Figure 3.**
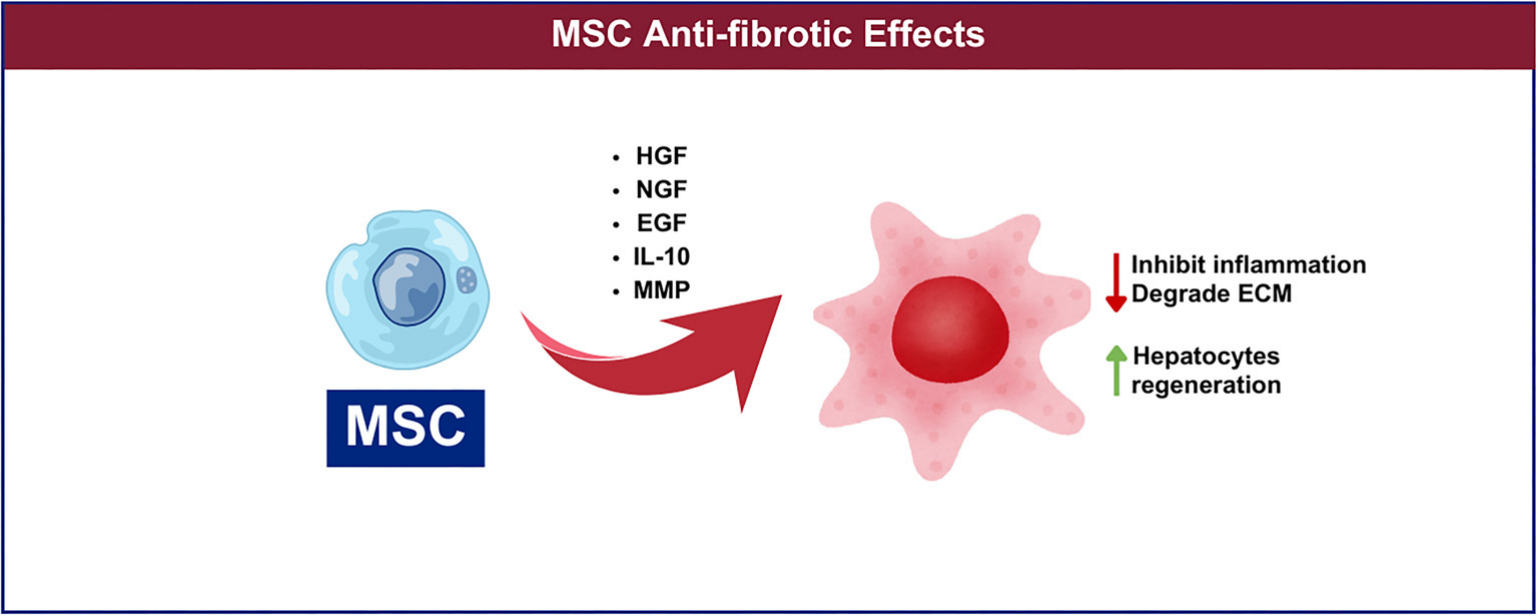
**MSC promotes anti fibrosis toward liver injury**. IL-10: Interleukin-10; MSC: Mesenchymal stem cell; MMP: Matrix metalloproteinase; HGF: Hepatocyte growth factor; NGF: Nerve growth factor; EGF: Epidermal growth factor; ECM: Extracellular matrix.

**Table 2:** Pathway cross-talk in MSC-mediated immunomodulation and anti-fibrosis

**Pathway**	**Role in immunomodulation**	**Role in anti-fibrosis**	**Shared/Unique**
TGF-β/Smad	Limited role in immunomodulation	MSCs inhibits the Wnt/β-catenin signaling pathway, thereby preventing the activation of HSCs and preventing liver fibrosis [70]	Shared
Wnt/β-catenin	Limited role in immunomodulation	MSCs inhibits the Wnt/β-catenin signaling pathway, thereby preventing the activation of HSCs and preventing liver fibrosis [71]	Unique to fibrosis
STAT3	MSCs regulate the STAT3 to promote the generation of Tregs and inhibit the release of pro-inflammatory factors, promote the polarization of macrophages toward the anti-inflammatory M2 phenotype, and reduce the pro-inflammatory M1 phenotype [72]	MSCs interfere with the activation of STAT3 by TGF-β, inhibit the activation of HSC, and suppress the synthesis of ECM components, such as COL1A1 and COL1A2 [73]	Shared
HGF	MSC-secreted HGF expands MDSCs, suppressing lymphocyte proliferation and increasing regulatory T cells to create an anti-inflammatory environment [74]	MSC-secreted HGF reduces HSC activation and collagen production, down regulates TGF-β1/Smad signaling pathway [75]	Shared
IL-10	Secreted by MSCs to suppress IFN-γ and IL-2 release, and cooperate with TGF-β to regulate immune microenvironment [76]	Secreted by MSCs to reduce HSC activation and collagen production [77]	Shared

MSC: Mesenchymal stem cell; TGF-β: Transforming growth factor-β; IL-10: Interleukin-10; STAT3: Signal transducer and activator of transcription 3; HGF: Hepatocyte growth factor; Tregs: Regulatory T cell; EC: Extracellular matrix.

### Anti-fibrosis effect

The liver is a fully regenerative organ; regenerative repair is typically complete after acute traumatic damage or minor tissue loss. However, when multiple chronic liver injuries occur, sustained damage activates HSCs, which are normally in a quiescent state. These activated HSCs begin secreting large amounts of type I and type III collagen fibers, leading to excessive extracellular matrix (ECM) deposition and fibrosis. If left untreated, this process can eventually progress to irreversible cirrhosis [66]. Some researchers used Transwell chambers to co-culture rat AD-MSCs with activated HSCs for 72 h. They found that the proliferative activity and collagen synthesis capacity of the HSCs were significantly reduced, while apoptosis was notably increased [67]. Various factors secreted by MSCs—such as hepatocyte growth factor (HGF), nerve growth factor (NGF), epidermal growth factor (EGF), IL-10, and matrix metalloproteinases (MMPs)—can inhibit inflammatory responses, degrade ECM, and promote endogenous hepatocyte regeneration [68]. Several animal studies have shown that transplantation of human umbilical cord-derived MSCs (HUC-MSCs) significantly reduces liver fibrosis and prevents its progression to cirrhosis, while also improving liver function and animal survival [69]. Figure 3 illustrates the overall anti-fibrotic effects of MSCs on liver injury. Despite involving distinct mechanisms, several signaling cascades have overlapping roles in both immunomodulation and fibrosis regulation. To better understand the interplay between these processes, the key pathways involved are summarized in Table 2. While some pathways—such as TGF-β/Smad—are shared between immunomodulation and anti-fibrosis, others, like Wnt/β-catenin, are more specific to fibrosis regulation.

**Figure 4.**
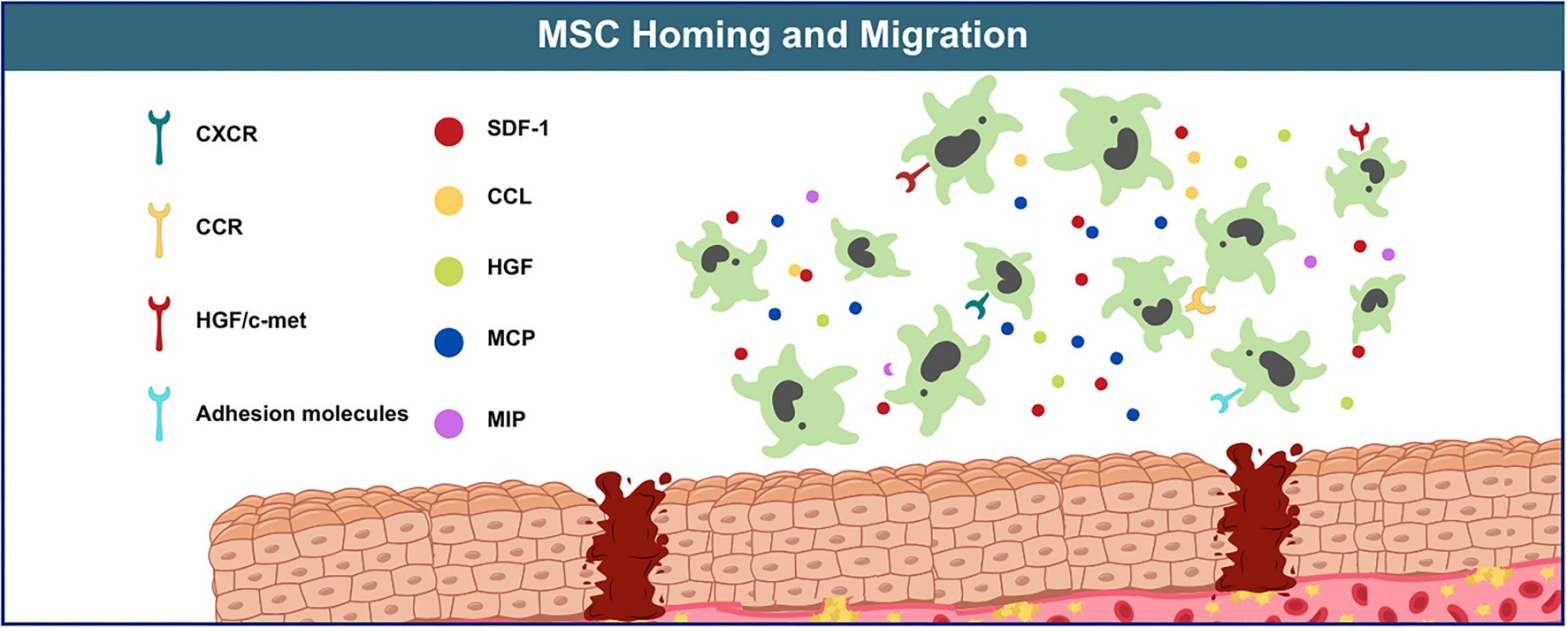
**Homing capability of MSC allows the migration of MSC to target tissue**. MSC: Mesenchymal stem cell; HGF: Hepatocyte growth factor; SDF-1: Stromal cell-derived factor 1; CXCR: CXC chemokine receptor; CCR: CC chemokine receptor; MCP: Monocyte chemotactic protein.

### Homing and migration

The “homing” of stem cells refers to their localization and retention within a specific microenvironment, known as the “stem cell homing environment.” This microenvironment regulates stem cell behavior, maintaining a balance between growth, renewal, and differentiation. The mechanism by which MSCs migrate across the endothelium and home to target tissues is not yet fully understood, but it likely involves the interaction of multiple receptors on the surface of MSCs—including chemokines, growth factors, adhesion molecules, and their corresponding ligands. Among the most studied are stromal cell-derived factor 1 (SDF-1), CXC chemokine receptor (CXCR), CC chemokine receptor (CCR), HGF and its receptor c-Met (HGF/c-Met), monocyte chemotactic protein (MCP), MIP, and various adhesion molecules. A schematic diagram is shown in Figure 4 [78]. This homing ability has been widely exploited by researchers to transplant MSCs directly into the injured liver microenvironment, where they can differentiate into hepatocytes and facilitate tissue repair. The number of MSCs that successfully home to the liver significantly influences their biological activity and therapeutic potential. Therefore, a better understanding of the homing mechanisms can enhance MSC efficacy in clinical applications by improving their homing efficiency. The route of transplantation plays a critical role in determining how many MSCs successfully colonize the liver, which in turn affects therapeutic outcomes. Common MSC transplantation routes for liver diseases include the peripheral vein, portal vein, intrahepatic, hepatic artery, intrapleural, and intraperitoneal routes [79]. Sang et al. [80] demonstrated that portal vein transplantation was superior for treating ALF, as it significantly improved liver function, reduced apoptosis, and extended survival. Additionally, intrahepatic injection has been shown to be particularly effective, as it minimizes the number of cells trapped in circulation and allows for a broader distribution throughout the liver parenchyma. In contrast, MSC-derived hepatocytes following intraperitoneal injection were primarily distributed around the portal vein [81]. Pretreatment of MSCs or optimization of their culture conditions can enhance the expression of homing-related molecules, thereby improving homing efficiency. Studies have shown that pretreatment with rapamycin or IL-1β increases MSC homing by upregulating CXCR4 expression, ultimately enhancing their therapeutic efficacy in ALF [82, 83]. Similarly, pretreating AD-MSCs with nitric oxide has been found to boost both their homing and proliferative capacities, improving outcomes in rat models of liver fibrosis [84]. Genetic modifications can also augment the therapeutic effects of MSCs, particularly through their exosomes. For instance, VEGF-165 gene modification improved MSC homing and colonization in liver tissue, reduced liver damage, and promoted regeneration in ALF rats [85]. Conversely, knockdown of miR-17 in MSCs abolished the therapeutic effects of their exosomes in ALF mice, indicating that miR-17 plays a critical role in the exosomes’ anti-inflammatory functions [86].

While homing is critical for MSC therapy, recent advances in enhancing exosome liver tropism have been explored to improve liver-targeting efficiency. A major challenge in exosome-based therapy is ensuring efficient delivery to liver tissue. To address this, strategies such as ligand engineering have been developed. For instance, Lin et al. (2024) demonstrated that engineering exosomes with CD44 ligands significantly enhances their uptake by hepatic cells, thereby improving their biodistribution and retention within the liver [87]. This approach leverages the interaction between CD44 and its ligand, hyaluronic acid (HA), which is highly expressed in liver endothelial cells and hepatocytes during injury. It presents a promising strategy to enhance MSC-Exo-based liver therapies, ensuring that therapeutic agents are effectively delivered to diseased liver tissue for the treatment of liver fibrosis and other liver-related disorders. In summary, MSCs are hypothesized to inhibit hepatic fibrosis and promote hepatocyte regeneration through various mechanisms, including differentiation, paracrine signaling, and the induction of HSC apoptosis. Although the specific regulatory pathways—such as how MSCs influence HSC activity, the mechanisms guiding MSC differentiation toward hepatocytes, and the factors affecting paracrine function—are not yet fully understood, MSC transplantation clearly offers a novel and promising approach for treating liver fibrosis.

## Results MSC and exosome-based therapies for specific liver diseases

In this section, we discuss the use of MSCs and MSC-Exos for treating various liver injuries, including HCC, acute and chronic hepatitis, acute liver failure, liver fibrosis and cirrhosis, alcoholic hepatitis, NASH, and hepatic ischemia-reperfusion (I/R) injury (Table 3). We also explore examples of different MSC sources and their therapeutic effects in liver disease models, as presented in Table 4. This table offers valuable insights into how MSCs from various origins may influence treatment outcomes in preclinical settings. Furthermore, Table 5 highlights clinical trial examples involving MSCs in the context of liver diseases, providing an overview of their real-world application and preliminary therapeutic results.

**Table 3:** Examples of the application of MSC-derived exosomes from different sources in liver diseases in pre-clinical model

**Disease**	**MSC**	**Application**	**Ref**
Acute liver injury	Mice BM-MSCs	MSC-Exo provides a protective effect against ferroptosis by maintaining SLC7A11 function	[88, 89]
Acute liver failure	HUC-MSCs	Reduces inflammatory cytokines and chemokine levels, decreases immune cell infiltration, and attenuates hepatocyte apoptosis	[90]
	Rat BM-MSCs	IL-1β pretreatment enhances MSC homing ability and improves therapeutic efficacy against ALF	[83]
	HUC-MSCs	Inhibits NLRP3 activation in macrophages and decreases proinflammatory cytokine levels	[91]
Liver fibrosis	HUC-MSCs	HSTP1-Exos target activated an HSC to enhance the therapeutic effect on liver fibrosis	[92]
	HBM-MSC-sEVs	Reduces liver fibrosis and collagen accumulation, enhances liver function, inhibits inflammation, and improves liver regeneration	[71]
	Peripheral blood MSCs	Inhibits HSC activation via the Wnt/β-Catenin signalling pathway	[93]
Hepatocellular carcinoma	AD-MSCs	Suppresses HCC by enhancing NKT cell anti-tumor responses	[94]
	HUC-MSCs	Inhibits the growth of liver cancer cells	[95]
	AD-MSCs	Exosomes from miR-122-modified AD-MSCs enhance chemosensitivity *in vitro* and *in vivo*	[96]

MSC: Mesenchymal stem cell; MSC-Exo: MSC-derived exosome; AD-MSC: Adipose-derived MSC; HUC: Human umbilical cord-derived MSC; HCC: Hepatocellular carcinoma.

**Table 4:** Examples of the application of different source MSCs and their therapeutic effects in liver diseases in pre-clinical model

**Disease**	**MSC**	**Application**	**Ref**
Acute liver injury	HUC-MSCs	Downregulate the IL-6, IL-1β, and TNF-α	[97]
	BM-MSCs	Suppress the expression of inflammatory cytokines & chemokines Inhibit NLRP3 inflammasome activation in hepatic macrophages Ameliorate liver inflammation by TAK1-NF-κB pathway	[98]
Liver fibrosis	BM-MSCs	Decrease in the face of inflammatory factors, such as IL-1, IL-2, IL-6, IL-8, IL-10, and TNF-α Prevent HSC activation and collagen deposition	[71]
	UC-MSCs	Inhibiting the TGF-β1/Smad signalling pathway	[99]
	AD-MSCs	Secreted high hepatocyte growth factor (HGF) Inhibited HSC secretion of type I collagen, MMP1, MMP2, and IL-6	[100, 101]
Alcoholic fatty liver disease	BM-MSCs	Prevent lipid accumulation, oxidative stress, and inflammation	[102]
	AD-MSCs	Reduced alcohol-induced damage, including lipid accumulation and fibrosis	[103]

TGF-β: Transforming growth factor-β; MSC: Mesenchymal stem cell; AD-MSC: Adipose-derived MSC; HUC-MSC: Human umbilical cord-derived MSC; MMP: Matrix metalloproteinase; IL-10: Interleukin-10.

**Table 5:** Examples of the application of MSCs in clinical trials in liver disease derivatives in acute liver injury and failure, highlighting key findings from relevant research

**Disease**	**MSC**	**Application**	**Ref**
Acute liver injury	UC-MSCs	Improvement in PTA and MELD score	[103]
Liver fibrosis	BM-MSCs	Improved liver function, including the MELD score, albumin levels, and coagulation function	[104]

MSC: Mesenchymal stem cell.

**Table 6:** Application of mesenchymal stem cells and their derivatives in acute liver injury and failure

**Disease**	**MSC Source**	**Exosome use**	**Findings**	**Ref**
Acute liver failure	HUC-MSCs	Applicable	Downregulate the IL-6, IL-1β, and TNF-α	[97]
Acute liver failure	BM-MSCs	Non-applicable	Suppress the expression of inflammator y cytokines & chemokines Inhibit NLRP3 inflammasome activation in hepatic macrophages Ameliorate liver inflammation by TAK1-NF-κB pathway	[98]
Hepatic ischemia-re perfusion injury	HUC-MSCs	HUC-MSC-EVs	Inhibitory effect on the chemotactic recruitment of neutrophil	[109]

MSC: Mesenchymal stem cell; HUC-MSC: Human umbilical cord-derived MSC.

### Acute liver failure and I/R injury

ALF is a clinical syndrome characterized by the rapid deterioration of liver function, leading to ascites, coagulopathy, hepatic encephalopathy, and multi-organ failure in patients without pre-existing liver disease [105]. ALF can result from severe drug-induced liver injury (DILI), hepatic ischemia, hepatotropic viral infections, or an aberrant immune response against foreign or self-antigens, triggered by hepatocyte injury [106]. In a mouse model of ALF, EVs secreted by either AD-MSCs or UC-MSCs downregulated inflammatory cytokines, such as IL-6, IL-1β, and TNF-α [97]. Wang et al. demonstrated that MSCs significantly suppressed the expression of inflammatory cytokines and chemokines in ALF. Additionally, MSC-derived PGE2 was shown to inhibit activation of the NLRP3 inflammasome in hepatic macrophages, thereby reducing liver inflammation. This occurs through suppression of the TGF-β-activated kinase 1 (TAK1)–NF-κB pathway [98]. Interestingly, Zhang et al. [107] found that the efficacy of MSC treatment in patients with liver failure was influenced by age. Hepatic I/R injury is a major cause of early postoperative hepatic insufficiency or liver failure. It commonly occurs after liver surgery, shock, trauma, organ transplantation, or acute massive blood loss. Contributing factors include oxidative stress, apoptosis, calcium overload, and, notably, the inflammatory response, which is considered a key driver of hepatic I/R injury [108]. MSCs from various sources—such as bone marrow, umbilical cord, and adipose tissue—have demonstrated significant therapeutic effects in mitigating hepatic I/R injury. MSCs reduce hepatocyte injury by modulating the liver’s inflammatory response and aiding in tissue repair. Furthermore, they inhibit the chemotactic recruitment of neutrophils, suggesting a role in attenuating liver damage by reducing neutrophil infiltration [109]. While the anti-inflammatory action of MSCs is a major therapeutic mechanism, additional pathways remain to be elucidated. Table 6 summarizes the applications of MSCs and their derivatives in acute liver injury and failure.

**Figure 5.**
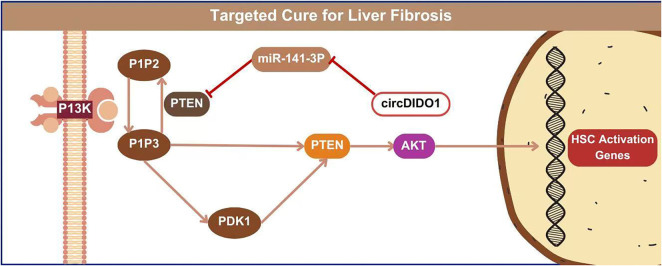
Comparing key signaling pathways, specifically PTEN/AKT in fibrosis.

### Hepatic fibrosis/cirrhosis

Viral liver fibrosis and cirrhosis are significant risk factors for the development of HCC. Therefore, slowing or halting the progression of liver fibrosis may help reduce the risk of malignant transformation. Reversal of liver fibrosis or cirrhosis can significantly decrease the incidence of end-stage liver disease. The biophysical and compositional properties of exosomes, along with their presence in biofluids, make them attractive candidates for blood-based liquid biopsy. Early assessment of the etiology of liver fibrosis is also crucial. HSCs transform into myofibroblasts, produce ECM proteins, and exacerbate oxidative stress and inflammation—processes that lead to notable changes in ECM quantity and composition [110]. Exosome-mediated HSC activation occurs through the release of inflammation-related factors, which activate biomarkers associated with ECM remodeling and promote liver fibrosis [111]. Hepatocyte-derived exosomes support hepatic homeostasis by promoting tissue repair and regeneration. In contrast, exosomes from stressed hepatocytes contribute to liver disease by inducing injury, inflammation, and fibrotic responses in recipient cells [112]. Wang et al. isolated NK cell-derived exosomes (NK-Exo) from NK-92MI cells and treated HSCs—major ECM-producing cells—with them. NK-Exo effectively inhibited TGF-β1-induced HSC proliferation and activation. Since HSC activation and transformation into myofibroblasts are central events in liver fibrosis, such interventions are significant [113]. Exosomes derived from HSCs themselves also contribute to fibrogenesis. These exosomes carry connective tissue growth factor (CCN2), which can be transferred between HSCs to amplify fibrogenic signaling [114]. As natural vehicles for targeted therapy, exosomes offer a precise delivery system for therapeutic agents and peptides aimed at HSCs, facilitating targeted treatment of liver fibrosis. For example, MSC-derived exosomes can deliver circDIDO1, which acts as a sponge for miR-141-3p, thereby inhibiting the PTEN/AKT pathway and suppressing HSC activation [115]. Figure 5 compares key signaling pathways, with a specific focus on the PTEN/AKT pathway in fibrosis. Similarly, exosomes from human bone marrow MSCs have been shown to alleviate liver fibrosis by inhibiting the Wnt/β-catenin pathway in HSCs [71]. These findings suggest that MSC-Exos can modulate key signaling pathways in HSCs to exert anti-fibrotic effects. Furthermore, exosomes from adipose tissue-derived MSCs engineered to overexpress miR-122 have been shown to more effectively suppress HSC activation and collagen deposition, indicating that miRNA modification may enhance the efficacy of exosome-based therapies [116]. Emerging bioengineering strategies, such as loading miRNAs into exosomes [117] or engineering exosomes to deliver CRISPR/Cas9 systems for therapeutic gene editing, are being actively explored for disease treatment [118, 119]. Among various therapeutic approaches, targeted therapy remains a popular focus. However, careful selection of therapeutic targets is crucial. For instance, TGF-β is a potent pro-fibrotic cytokine that drives HSC activation and survival, making it a key candidate for anti-fibrotic intervention [120].

During the progression of liver fibrosis, the accumulation of inflammatory factors in the liver enhances the hepatic inflammatory response, causing further liver damage. Inflammation is a key factor in both the initiation and maintenance of liver fibrosis [121]. In one study, BM-MSCs were transplanted into rats with CCl_4_-induced liver fibrosis. Quantitative real-time PCR (qRT-PCR) was used to detect the expression of inflammatory factors in liver tissue, and a significant decrease was observed in the levels of IL-1, IL-2, IL-6, IL-8, IL-10, and TNF-α following treatment [76]. The TGF-β1/Smad signaling pathway plays a crucial role in liver fibrosis [122]. Elevated levels of the inflammatory factor TGF-β1 increase hepatic inflammation, promote HSC activation, and contribute to collagen deposition [123]. The Smad protein family is central to the TGF-β1 pathway and acts as a key regulator of hepatic fibrosis [124]. Yi et al. demonstrated that microencapsulated hUC-MSCs, constructed by transfecting alginate-polylysine-alginate (A-P-A) microcapsules with HGF, and transplanted into rats with CCl_4_-induced liver fibrosis, significantly ameliorated fibrosis. This effect was closely associated with inhibition of the TGF-β1/Smad signaling pathway [99]. Further, the same team showed that HGF-transfected HUC-MSCs reduced the viability of HSC-T6 cells, promoted apoptosis, inhibited activation, and decreased the expression of collagen I, collagen III, TGF-β1, Smad2, and other Smad proteins. These results suggest that the anti-fibrotic effect of HUC-MSCs may be mediated through inhibition of the TGF-β1/Smad pathway [122]. MSCs also exert anti-fibrotic effects through immune modulation. Luo et al. showed that BM-MSCs transplanted into mice via the tail vein suppressed proinflammatory M1 macrophage activation. This led to decreased expression of IFN-γ, TNF-α, and IL-6, resulting in reduced HSC-mediated fibrogenesis. Further findings indicated that BM-MSC transplantation not only inhibited M1 macrophage activation but also promoted M2 macrophage polarization and increased MMP13 expression—both of which contribute to HSC inhibition and anti-fibrotic effects [125]. In another experiment, human hepatic MSCs co-cultured with HSCs were found to secrete high levels of HGF, a growth factor with anti-fibrotic properties [100]. Analysis of HSC proliferation using flow cytometry, immunocytochemistry, ELISA, and Luminex assays revealed that HGF inhibited the secretion of type I collagen, MMP1, MMP2, and IL-6 by HSCs. This, in turn, reduced ECM deposition and improved liver function, suggesting that HGF may be a promising therapeutic target for liver fibrosis [101]. MSCs also act through homing mechanisms. In a study by Wu et al., AD-MSCs injected into CCl_4_-treated mice via the tail vein showed significant liver fibrosis improvement after three weeks. The AD-MSCs were able to migrate to the liver, survive, and differentiate into hepatocytes [126]. Similarly, Chai et al. infused green fluorescent protein-labeled UC-MSCs into rats with liver injury and fibrosis. The UC-MSCs localized to the damaged liver tissue alongside infiltrating inflammatory cells, differentiated into hepatocytes, and promoted liver recovery [127]. Further analysis using phagocytosis assays and flow cytometry showed that UC-MSCs promoted the conversion of Kupffer cells (resident liver macrophages) into anti-inflammatory M2 macrophages, thereby reducing the release of inflammatory cytokines and mitigating liver inflammation. Several clinical trials have evaluated the safety and efficacy of MSC therapy in liver failure and cirrhosis [37]. A phase I trial using UC-MSCs in patients with liver failure demonstrated safety and improvement in liver function, including reduced bilirubin and ALT levels and increased survival, although the long-term efficacy remains uncertain. In patients with decompensated cirrhosis, a phase I–II trial involving four patients confirmed the safety and feasibility of MSC therapy and reported improvements in quality of life. Additional trials in HBV/HCV-induced cirrhosis showed partial liver function improvements, though some did not find significant benefits [128]. Larger, well-designed clinical trials are necessary to confirm the therapeutic potential of MSCs across different liver disease types. Table 7 below summarizes the key findings on the use of MSCs and their exosomes in treating liver fibrosis and cirrhosis.

**Table 7:** Application of mesenchymal stem cells and their exosomes in liver fibrosis and cirrhosis

**Disease**	**MSC source**	**Exosome use**	**Findings**	**Ref**
Liver fibrosis	BM-MSCs	MSC derived exosome	Alleviate liver fibrosis by inhibiting the Wnt/β-caten in pathway in HSCs Inhibiting the PTEN/AKT pathway and suppressing HSC activation	[71, 115]
Liver fibrosis	AD-MSCs	Overexpress ion of miR-122 in exosomes	Effectively suppressed HSC activation and ECM deposition, showing improved anti-fibrotic effects	[116]
Liver fibrosis	UC-MSCs	Non applicable	Inhibiting the TGF-β1/Smad signalling pathway	[99]
Liver fibrosis	BM-MSCs	Non applicable	Suppression of proinflamma tory M1 macrophage activation Reduced the expression of inflammatory factors, such as IFN-γ, TNF-α, and IL-6	[125]
Liver fibrosis	AD-MSCs	Non applicable	Anti-liver fibrosis	[126]
Liver fibrosis	UC-MSCs	Non applicable	Prompted the conversion of Kupffer cells (liver resident macrophages) into M2 macrophages Reducing the release of inflammatory factors and attenuating the degree of liver inflammation	[127]

MSC: Mesenchymal stem cell; TGF-β: Transforming growth factor-β; AD-MSC: Adipose-derived MSC; ECM: Extracellular matrix.

### HCC

Stem cell exosomes have shown significant potential in the treatment of liver cancer. Exosomes derived from UC-MSCs have demonstrated anti-proliferative, pro-apoptotic, and anti-angiogenic effects on HCC cells. These exosomes inhibit HCC cell survival and downregulate the expression of oncogenes associated with HCC progression [129]. Moreover, exosomes from MSCs have been investigated as delivery systems for microRNA-based therapies, showing promising results in suppressing HCC cell proliferation, invasion, and metastasis [130]. Gu et al. used MSC-Exos to effectively inhibit the malignant behavior of HCC tumor stem cells (CSCs) by regulating the C5orf66-AS1/miR-127-3p/DUSP1/ERK pathway, thereby highlighting their therapeutic potential in targeting CSC stemness [131]. Lou et al. studied the effects of AD-MSCs modified with miR-199a. The engineered exosomes (AD-MSC-Exo-199a) significantly enhanced the chemosensitivity of HCC cells by targeting the mTOR pathway, improving the delivery efficiency of adriamycin (Dox) to HCC cells, and demonstrating therapeutic efficacy *in vivo* [132]. A disintegrin and metalloproteinase 10 (ADAM10) plays a critical role in modulating HCC cell chemosensitivity and serves as a target for improving chemotherapy outcomes [133]. Xu et al. found that miRNA-451a, delivered via human UC-MSC-Exos, inhibited epithelial–mesenchymal transition (EMT)-related proteins and paclitaxel resistance, promoting apoptosis in HCC cells by targeting ADAM10 [134] BM-MSCs can integrate into tumor tissues. When engineered to overexpress TNF-related apoptosis-inducing ligand (TRAIL), these MSCs can deliver TRAIL directly to tumors, significantly reducing tumor growth [135]. Although TRAIL is a promising anti-cancer agent, drug-loaded MSCs can cause toxicity to non-target tissues, necessitating careful consideration of drug concentration, loading capacity, and cell number [136]. Due to the limited drug-carrying capacity of MSCs, researchers have increasingly turned to MSC-Exos, which retain the biological functions of MSCs but are smaller, capable of crossing biological membranes, and exhibit low immunogenicity [137]. Deng et al. [138] demonstrated that BM-MSC-Exos promote apoptosis in HCC cells by delivering miRNA-20a-3p, which targets apoptosis inhibitory proteins and upregulates TRAIL expression, further enhancing apoptosis. In xenograft models, TRAIL-overexpressing MSCs increased miRNA-7 levels, promoted apoptosis, and inhibited tumor growth via an exosome-dependent mechanism, with miRNA-7 identified as a key sensitizer of TRAIL-induced apoptosis [139]. GRP78 is overexpressed in cancer cells resistant to the chemotherapeutic drug sorafenib. In one study, MSC-Exos modified with si-GRP78 were shown to bind sorafenib, target GRP78 in cancer cells, and suppress growth, invasion, and metastasis both *in vitro* and *in vivo*, thereby reversing sorafenib resistance [63]. Table 8 below summarizes the applications and findings of MSC-Exos in HCC therapy.

**Table 8:** Application of mesenchymal stem cell-derived exosomes in hepatocellular carcinoma therapy

**Disease**	**MSC source**	**Exosome use**	**Findings**	**Ref**
Hepatocellular carcinoma	UC-MSCs	UC-MSC exosome	Anti-proliferative, apoptotic, and anti-angiogenic effects	[129]
Hepatocellular carcinoma	BM-MSCs	BM-MSC exosome	Block malignant behaviors of HCC-sourced CSCs through a C5orf66-AS1 /miR-127-3p /DUSP1/ERK axis	[131]
Hepatocellular carcinoma	AD-MSCs	ADMSC-Exo-1 99a	Increased chemosensitivity of HCC cells through targeting mTOR pathway Improved adriamycin (Dox) delivery efficiency to HCC cells	[132]
Hepatocellular carcinoma	UC-MSCs	UC-MSC-Exo miRNA-451a	Inhibited epithelial–mesenchymal transition (EMT)-related proteins and paclitaxel resistance Promoting apoptosis in HCC cells by inhibiting ADAM10	[134]
Hepatocellular carcinoma	BM-MSCs	BM-MSC-Exos	MiR-20a-3p: Targets c-FLIP, increases TRAIL level to promote HCC apoptosis	[138]
Hepatocellular carcinoma	BM-MSCs	si-GRP78-modified MSC-Exos	Inhibits growth, invasion, and metastasis	[63]

MSC: Mesenchymal stem cell; MSC-Exos: MSC-derived exosomes; AD_MSC: Adipose-derived MSC; HCC: Hepatocellular carcinoma; ADAM10: A disintegrin and metalloproteinase 10; TRAIL: TNF-related apoptosis-inducing ligand.

### NAFLD/NASH

NAFLD is a nonalcoholic, genetically, environmentally, and metabolically stress-induced liver disease characterized primarily by lipid accumulation in the liver. It is the most common cause of liver disease worldwide. Recently, the progression of NAFLD to cirrhosis and the incidence of related complications have increased. Exosomes are involved in both the pathogenesis and progression of NAFLD, contributing to inflammation and fibrosis—key processes in the transition from isolated steatosis to NASH. Data from animal models of diet-induced NASH suggest that exosome concentrations increase over time as the disease progresses. This increase appears to be a response to the accumulation of toxic lipids and their downstream mediators in the liver, which enhance hepatocytes’ capacity to produce and release various types of exosomes [140]. Lipid toxicity and macrophage-mediated inflammation are critical factors in the development of NASH. Gu et al. developed a NASH animal model using a high-fat diet (HFD) combined with methionine–choline deficiency (MCD). Their findings indicated that endoplasmic reticulum stress prompts adipocytes to secrete exosomes containing aldose reductase 1B7 (Akr1B7), which are then taken up by hepatocytes. This uptake increases glycerol levels in hepatocytes and promotes the development of NASH, implicating AKR1B7 in disease progression [141]. In contrast, alcoholic fatty liver disease results from alcohol-induced hepatocyte necrosis. BM-MSC transplantation has been shown to significantly alleviate alcohol-induced liver damage in mice, including lipid accumulation, oxidative stress, and inflammation [102]. Similarly, transplantation of human AD-MSCs effectively reduced CYP2E1 expression, increased the activity of the acetaldehyde-metabolizing enzyme ALDH2, and mitigated alcohol-induced damage, such as lipid accumulation and fibrosis [142]. Based on promising preclinical findings, MSCs have been administered to patients with alcoholic cirrhosis.

### DILI

DILI is a significant medical concern due to its unpredictable nature and potential severity, ranging from mild biochemical abnormalities to acute liver failure. The pathogenesis of DILI involves various mechanisms, including mitochondrial injury, immune responses, and genetic polymorphisms in drug-metabolizing enzymes. Antimicrobials and herbal supplements are common culprits [143]. MSCs and their derived exosomes have emerged as promising therapeutic approaches for DILI. Exosomes derived from HUC-MSCs have been shown to mitigate acetaminophen (APAP)-induced hepatocyte damage, enhance cell viability, and restore redox balance and aminotransferase activity in APAP-treated cells [144]. Additionally, MSC-Exos attenuate CCl_4_-induced liver injury in mice, as evidenced by increased levels of proliferating cell nuclear antigen (PCNA) and cyclin D1 expression. These exosomes also inhibit APAP- and hydrogen peroxide (H_2_O_2_)-induced hepatocyte apoptosis by upregulating Bcl-xL protein expression [145].

## Discussion

One of the foremost challenges in the field of medical science, particularly in MSC studies and their clinical applications, is the lack of standardization across cell and gene therapies. Variability in cell sourcing, culture conditions, and MSC characterization across different facilities can result in inconsistent findings, making it difficult to compare studies and draw definitive conclusions about MSC efficacy [146]. Ensuring the safety of MSCs—particularly concerns around tumorigenicity and potential alterations to immune responses—remains an ongoing issue, despite regulatory oversight [147]. Additionally, challenges around dosing and scalability for large-scale manufacturing persist. Optimal dosing strategies—including the number of cells, timing, and frequency of administration—have yet to be fully established. Furthermore, large-scale production requires rigorous quality control to ensure reproducibility and safety, which adds complexity to the regulatory approval process [148]. The U.S. Food and Drug Administration (FDA) has recently emphasized the importance of engineering and quality control in the biomanufacturing of EV-based products, including those derived from MSCs. The FDA highlights the need for advanced analytical tools to assess EV identity, quantity, and functionality, and stresses the importance of process optimization to ensure batch-to-batch consistency. These guidelines aim to address the safety and clinical efficacy of EV-based therapies, including tumorigenicity risks, and to provide a framework for advancing such products toward clinical translation and regulatory approval. As of 2020, more than 150 clinical trials related to EVs were ongoing (ClinicalTrials.gov). From May 29, 2020, to January 25, 2022, a project led by Steven M. Jay (University of Maryland, College Park) and Steven R. Bauer (FDA) sought to bridge the quality control gap for MSC-derived EVs. Therefore, manufacturing MSC and exosome products in facilities compliant with current good manufacturing practices (cGMP), and accredited by regulatory authorities, is critical for producing high-quality, consistent MSC therapies [149]. Although producing MSC and exosome products in cGMP facilities can be costly, labor-intensive, and time-consuming, prioritizing safety and quality is essential for developing reliable stem cell therapies for human use [150]. Several knowledge gaps remain in our understanding of MSC biology and therapeutic mechanisms. For instance, fine-tuning the cargo of MSC-Exos for targeted therapies is still in its early stages. Current research is focused on controlling and modifying the content of exosomes released by MSCs to enhance their therapeutic potential [151]. Moreover, there is limited understanding of the conditions—such as the cytokines, growth factors, and microenvironment—needed to direct MSC differentiation into specific cell types (e.g., partial vs full hepatic differentiation for liver repair) [152]. Gaining deeper insight into these fundamental biological processes is essential for optimizing MSC therapies and broadening their clinical applications.

In terms of clinical translation, Phase III/IV clinical trials remain insufficient, which hampers the validation of MSC therapies for broader medical use. Additionally, determining the optimal route of administration—whether local or systemic—may influence treatment or regenerative efficacy, as it depends on the homing capability of MSCs to the target region [44]. A recent preclinical study in BALB/c mice using human bone marrow-derived MSCs demonstrated short- and long-term safety, with no abnormal immune responses, organ damage, or toxicity observed [153]. However, clinical data on the safety of short- and long-term treatments using different MSC sources and transplantation methods remain limited. Although a meta-analysis of 62 randomized clinical trials involving 3,546 participants confirmed the safety of MSC therapy across various populations compared to placebo [154], additional safety data are still needed to support the exploration of these treatments across a wider range of diseases. Exosome heterogeneity and batch variability present significant challenges to achieving consistent therapeutic outcomes in regenerative medicine and disease therapy. Similar to MSCs, exosome heterogeneity arises from variables, such as cell source, physiological condition, cargo composition, and size—all of which affect biological function. These factors can result in variations in the composition, size, and function of secreted exosomes [155, 156]. Batch variability may be influenced by isolation techniques, culture conditions, storage stability, and donor cell differences [157]. Different isolation methods yield exosomes with varying purity, size, and biological activity, while even minor changes in culture or storage conditions can affect their potency. These inconsistencies impact therapeutic reliability, with some exosomes promoting tissue repair while others do not—leading to variability in efficacy, safety, and reproducibility. Moreover, regulatory and scalability issues present additional obstacles in the clinical application of exosome-based therapies. Regulatory bodies such as the FDA and EMA enforce stringent safety, efficacy, and quality standards; however, the lack of standardized protocols for exosome isolation, purification, and characterization complicates approval processes [157]. Inconsistencies in production methods further hinder the ability to meet regulatory requirements due to variations in exosome composition and potency. Future efforts should focus on addressing these challenges through more rigorous clinical trials evaluating MSC therapy for liver disease, development of standardized regulatory frameworks, and advancements in biomanufacturing technologies. By closing the current gaps in both foundational science and clinical practice, MSC-based therapies may become a viable option for a broad spectrum of conditions, ranging from wound healing to chronic diseases such as liver disease.

## Conclusion

MSCs are a safe and promising therapeutic strategy currently being evaluated in clinical trials for the treatment of liver diseases, such as cirrhosis and liver failure caused by HBV, HCV, or alcohol-related etiologies, as well as complications following liver transplantation. However, MSCs represent a heterogeneous cell population, and several critical issues must be addressed before their widespread therapeutic application. First, there is a lack of standardized criteria for evaluating MSC quality across trials, including the stability of cells cultured long-term *in vitro*. Improved standardization in the isolation and characterization of exosomes would also enhance the reliability of results [157]. More clinical trials, especially phase III randomized controlled trials, are needed to establish the efficacy, safety, optimal dosage, and to monitor potential side effects of this treatment modality. Second, there is insufficient research on optimal treatment parameters, such as timing, cell dose, administration intervals, cell source, minimum effective cell number, and delivery route. These aspects require further investigation to maximize therapeutic efficacy while balancing cost and feasibility. Finally, the mechanisms by which MSCs mediate liver regeneration remain poorly understood. Key processes—including the activation environment of MSCs, cytokine secretion responsible for anti-inflammatory, antioxidant, and anti-fibrotic effects, and the involved signaling pathways—warrant further study. The therapeutic mechanisms of MSCs from various sources have yet to be clearly and comprehensively elucidated. Future research should address these gaps through long-term clinical studies aimed at developing standardized protocols and optimizing MSC-based therapies for liver disease. Compared with MSCs, MSC-Exos offer unique advantages: (1) they are easier to collect, as MSCs can secrete them in large quantities; (2) their small size allows for stable long-term storage; and (3) they are non-proliferative and non-tumorigenic in clinical applications, making them safer than MSCs. Due to some of the limitations of MSCs, exosomes are considered more promising as carriers for drugs or genes. MSCs and their exosomes also hold significant potential as drug delivery systems for cancer therapy. However, further research is necessary to fully understand their therapeutic mechanisms and realize their potential in exosome-based treatments. Despite their promise, exosomes are associated with certain risks and limitations, such as immune reactions, unclear long-term safety, off-target effects, and possible prothrombotic complications. Ongoing research aims to mitigate these issues, and rigorous standardization, purification, and quality control of exosome therapies will be crucial to minimizing adverse effects.
